# Oxidation of 5‐Hydroxymethylfurfural into 2,5‐Diformylfuran on Alkali Doped Ru/C Catalysts. Electron Properties of Ruthenium Species as Descriptor of Catalytic Activity

**DOI:** 10.1002/cssc.202401515

**Published:** 2024-10-25

**Authors:** Beatriz Hurtado, Karen S. Arias, Patricia Concepción, Maria J. Climent, Sara Iborra, Avelino Corma

**Affiliations:** ^1^ Instituto de Tecnología Química Universitat Politècnica de València- Agencia Estatal Consejo Superior de Investigaciones Científica Avda dels Tarongers s/n Valencia 46022 Spain

**Keywords:** Alkali doped carbon, Biomass, Oxidation, Ruthenium catalyst, 2,5-diformylfuran, 5-hydroxymethylfurfural

## Abstract

Selective aerobic oxidation of 5‐hydroxymethylfurfural into 2,5‐diformylfuran has been achieved on alkali doped Ru/C catalyst. Optimization of Ru metal nanoparticles, as well as the nature and amount of the alkali dopant have been performed. The results showed that doping the Ru/C catalyst with controlled amount of potassium increases the catalytic activity, 2.5 fold with respect to the non‐doped sample. Spectroscopic studies showed that these differences in activity can be attributed to a different oxidation reaction mechanism associated to the presence of electron rich Ru species in the promoted sample that facilitate the dissociation of O_2_, while prevents the oxidation of the metal. The Ru/C−K doped catalyst resulted very stable against leaching and metal sintering, being possible the reuse over several consecutive runs. Moreover, the catalyst could be successfully applied to the oxidation of different alcohols.

## Graphical abstract

5‐Hydroxymethylfurfural is selectively oxidized into 2,5‐diformylfuran by using nanoparticles of ruthenium supported on potassium doped active carbon. The catalyst exhibits increased catalytic activity and stability compared to undoped Ru/C. Spectroscopic studies indicate that this behaviour is associated with the presence of electron‐enriched Ru species that facilitate the dissociation of O_2_ and, at the same time, prevent metal oxidation.

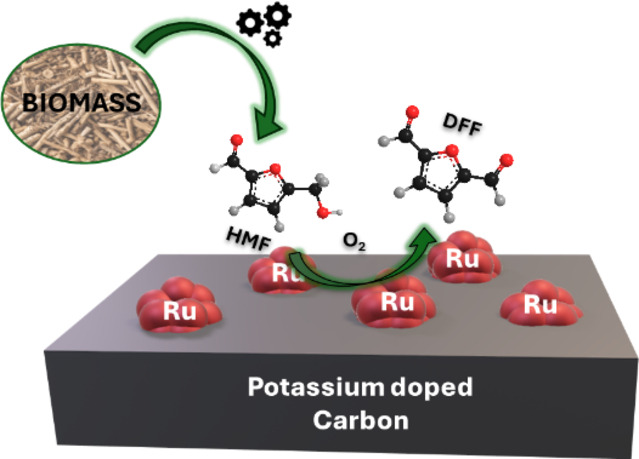

## Introduction

**Scheme 1:**
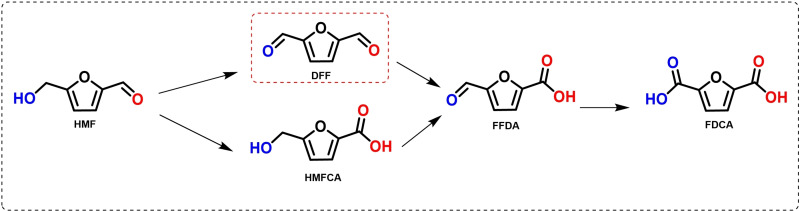
Oxidation reaction of HMF into different products.

The transformation of platform molecules derived from biomass into biofuels and high‐value chemical products constitute an important alternative to fossil resources[^1^, ^2^] to mitigate climate change and global warming. 5‐Hydroxymethylfurfural (HMF), derived from the acid catalyzed dehydration of hexoses represents one of most versatile platform molecules that can be converted into a wide variety of compounds of interest through different catalytic pathways.[^3^, ^4^, ^5^, ^6^, ^7^, ^8^] During the last decades, the oxidation reaction of HMF into a variety of high added value compounds, such as 2,5‐furandicarboxylic acid (FDCA),^[9]^ 5‐formyl‐2‐furancarboxylic acid (FFCA),^[10]^ 5‐hydroxymethyl‐2‐furancarboxylic acid (HMFCA),^[11]^ and 2,5‐diformylfuran (DFF) has been extensively studied.^[12]^ Among them, DFF is a versatile precursor for manufacturing organic conductors, cross‐linking reagents, pharmaceuticals, and as monomer for preparation of many polymers, etc.[^13^, ^14^, ^15^, ^16^, ^17^, ^18^] However, the selective oxidation of the hydroxymethyl group of HMF to obtain 2,5‐diformylfuran (DFF) is not a simple process, since in many cases it can undergo many side reactions as over oxidation of the aldehyde groups and cross‐polymerization leading to a mixture of products with the corresponding low selectivity to DFF (Scheme 1). In the last years the oxidation of HMF to DFF has been extensively studied over homogeneous and metal (noble and no noble) supported heterogeneous catalysts such as Pd, Pt, Au, Ru, V, Mn, Cu, Co or Fe, but most of the existing catalytic systems are still requiring relatively harsh reaction conditions (high temperature and pressure) and the use of harmful organic solvents. The topic has been recently reviewed by Tran^[23]^ and Ju et al.^[24]^ Among the different metals used in the oxidation of HMF into DFF, Ru performs very well in terms of activity and selectivity to DFF and therefore Ru nanoparticles loaded over different supports such as activated carbon, metal oxides or polymeric materials have been reported (see Table S1). Additionally, several studies using simple metal oxides with no auxiliary metal such as MnO_2_,[^19^, ^20^] have showed high selectivity and yield to DFF, although the turnover frequency are several orders of magnitude lower compared to the use of metal‐based catalysts. Also, it is worth noting the recently reported selective oxidation of HMF into DFF through electrochemical methods[^21^, ^22^] which represents a sustainable strategy of synthesis.

Among the different materials used as supports for metal and metal oxides, the abundant, readily available and cheaper activated carbon meet all the requirements to be used at a commercial scale.^[25]^ Particularly, for the oxidation of HMF into DFF Nie et al.^[26]^ reported that activated carbon‐supported Ru clusters (Ru/C) efficiently catalyzed HMF oxidation to DFF with 96 % yield at 110 °C and 20 bar O_2_ in toluene.

On the other hand, it is known that the doping of metal heterogeneous catalysts with alkali or alkaline earth metals (K, Cs, Ba, Na) can increase the activity and/or selectivity for some metal‐catalyzed reactions[^27^, ^28^, ^29^, ^30^] such as ammonia synthesis,[^31^, ^32^, ^33^, ^34^, ^35^, ^36^] NO oxidation,^[37]^ hydrogenation reactions,[^38^, ^39^] etc. Aika et al. studied the effect of the doping with alkali metals or their oxides of a variety of Ru catalyst supported on activated carbon and on various inorganic carriers for the synthesis of ammonia.[^40^, ^41^] The authors concluded that the promoter effect is provided by a charge transfer from the alkali metal to Ru, which results in a higher electron density in the transition metal,^[42]^ favouring the activation of nitrogen. Moreover, they found that, among the different supports, active carbon and alumina exhibited the highest specific reaction rates per surface ruthenium, which suggests a role of these materials on the final properties of RuO_2_.

Considering these precedents, and with the aim to develop a simple, cheap and scalable catalyst to produce selectively DFF from the aerobic oxidation of HMF, we have studied the effect of doping with alkali salts (Na, K, Cs) a Ru/C catalyst on the activity, selectivity and catalyst stability. We will show that an optimized catalyst based on Ru nanoparticles supported on potassium doped activated carbon, increases the catalytic activity by a 2.5 fold with respect to the non‐doped Ru/C, while keeping stable when used in consecutive cycles. The higher activity and stability exhibited by the potassium promoted Ru/C catalyst with respect to the non‐promoted sample, is mainly attributed to a different oxidation reaction mechanism associated to the presence of electron rich Ru species that facilities the dissociation of O_2_, while prevents the oxidation of the metal, as determined by spectroscopic studies.

## Results and Discussion

### Oxidation of HMF into DFF

For this study, we first evaluate the activity and selectivity of different Ru supported catalysts in the oxidation of 5‐HMF to DFF (Table 1). The reactions were performed under previously optimized conditions at 120 °C and 6 bar of O_2_ using trifluorotoluene (TFT) as a solvent. As can be observed, commercial 5 % Ru/Al_2_O_3_, 3 % Ru/HT, 3 % Ru/TiO_2_, 3 % Ru/ZrO_2_ catalysts gave maximum selectivity to DFF although low HMF conversion while the commercial 5 % Ru/C exhibited the highest performance in terms of conversion and yield of DFF although ~8 % overoxidation products were also detected which slightly decreases the DFF selectivity (91 %).

**Table 1:** Oxidation of 5‐hydroxymethylfurfural with different Ru catalysts.^[a]^

Entry	Catalyst	Conv. HMF (%)	Yield DFF (%)	Yield FFCA (%)	Yield HMFCA (%)	Yield FDCA (%)	Selec. DFF (%)
1^[b]^	5 % Ru/Al_2_O_3_ com.	46	46	–	–	–	100
2^[b]^	5 % Ru/C com.	99	90	2.5	5.5	1	91
3	3 % Ru/HT^[c]^	29	29	–	–	–	100
4	3 % Ru/TiO_2_	64	64	–	–	–	100
5	3 % Ru/ZrO_2_	33	33	–	–	–	100
6	3 % Ru/C	99	99	–	–	–	100

[a] Reaction conditions: HMF (0.5 mmol), HMF/Ru (molar)=84, 6 bar O_2_, 120 °C, 1000 rpm, 6 h. [b] Commercial catalyst from Sigma‐Aldrich. [c] Al−Mg mixed oxide derived from hydrotalcite.

### Influence of the Ru Crystal Size on Catalytic Activity

Considering that Ru/C display the highest efficiency on the HMF oxidation reaction, the next step was to optimize the size of nanoparticles of Ru on active carbon.

**Figure 1:**
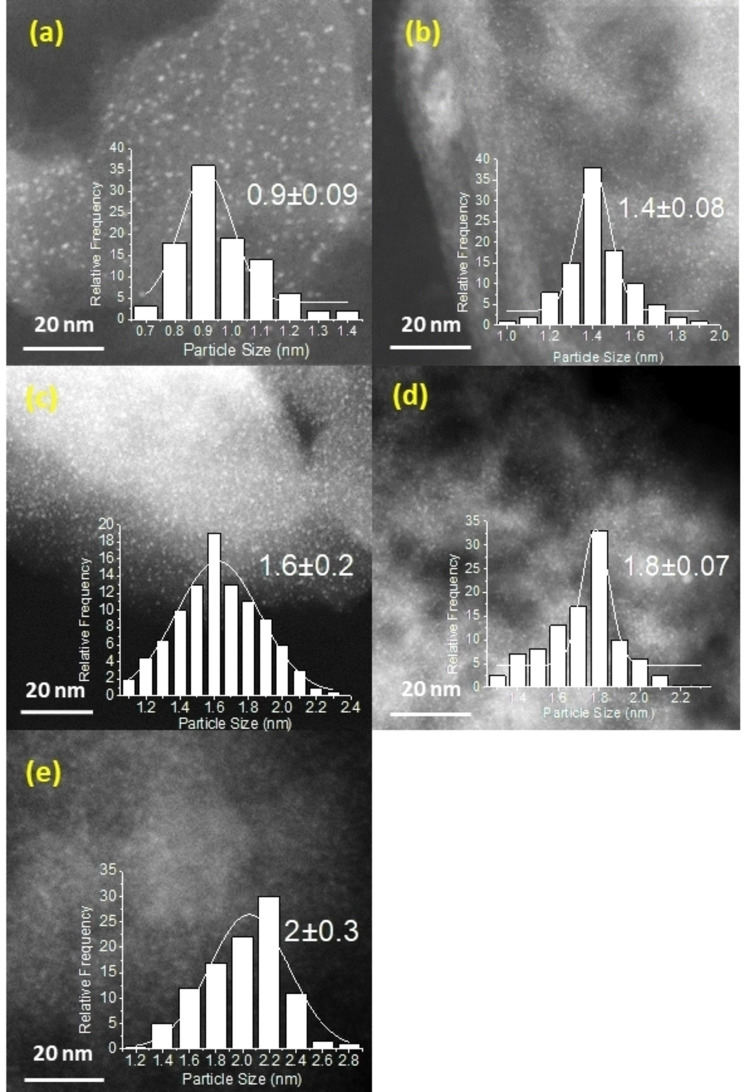
STEM images of the catalysts (a) 0.5 % Ru/C, (b) 1 % Ru/C, (c) 3 % Ru/C, (d) 6 % Ru/C (e) 10 % Ru/C.

To study the influence of crystal size of Ru nanoparticles on the activity and selectivity we have modified the metal crystallite size by changing the amount of Ru on the support by preparing a series of samples with different Ru loadings ranging from 0.5 to 10 wt % of Ru on activated carbon. The amount of ruthenium was measured by X‐ray fluorescence spectroscopy (see Table S2). The particle size distribution of the Ru nanoparticles was determined by Transmission Electron Microscopy (TEM) and scanning transmission electron microscopy (STEM). As can be observed in the histograms (Figure 1), the average size of Ru nanoparticles and the distribution range increases when increasing the percentage of Ru on the support.

**Figure 2:**
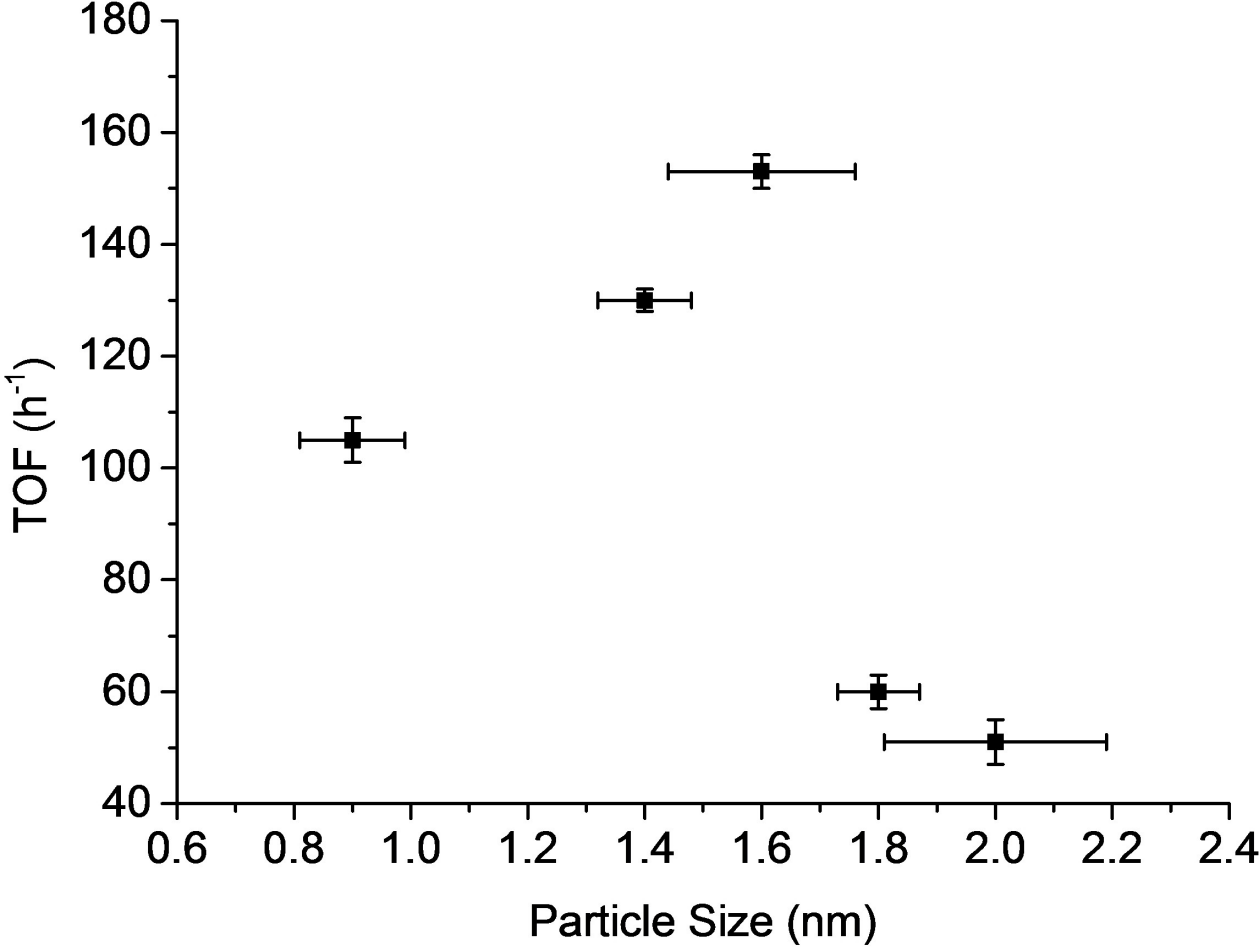
TOF versus particle size measured by STEM.

The samples were tested in the oxidation of HMF (Table 2), previous reduction at 400 °C in a H_2_ atmosphere, according to TPR measurements (Figure S1). As can be observed, maximum selectivities were achieved in all cases (kinetic plots are displayed in Figure S2). However, the maximum activity was exhibited by the sample 3 %Ru/C (with an average crystal size of 1.6 nm) in terms of initial reaction rate and conversion. The impact of particle size can be investigated by calculating the turnover frequency (TOF) and how it changes with particle size. The TOF of the different samples were calculated from the initial reaction rate of HMF disappearance divided by the number of accessible Ru measured by CO chemisorption (see experimental section). The plot of TOF of the catalysts versus particle size (Figure 2) showed a volcano curve, reaching maximum activity at 1.6 nm is observed. This indicates that the oxidation of HMF is a reaction sensitive to the size of the metal nanoparticle.^[43]^ The structure sensitivity of Ru supported catalysts has also been previously observed for ammonia synthesis,^[44]^ where it was suggested that B5‐type sites (an active site that consists of 5 atom surfaces located at the edges on small Ru crystals with only hcp (001) and (100) surfaces exposed), and which proportion follows a volcano curve with the crystal size, are the main responsible for the catalytic activity. Similar results were also reported by Regalbuto et al.^[38]^ in the hydrogenation of levulinic acid into g‐valerolactone using Ru supported catalysts with Ru crystal sizes ranging from 0.9 to 2.5 nm, and where the volcano peak (of the curve of TOF versus crystal size) was encountered at 1.5 nm. In our case, the presence of B5‐type sites on Ru/C samples could not be determined by FTIR of CO adsorbed on Ru nanoparticles due to the carbon support, however considering that the amount of surface Ru species determined by CO chemisorption is rather similar among the different samples, the different catalytic activity encountered for the different nanoparticles surfaces point to a similar particle size reliance.

**Table 2:** Oxidation of 5‐hydroxymethylfurfural with different loadings of Ru/C catalysts.^[a]^

Entry	Catalyst	Ru content (%wt)^[b]^	Average Particle Size (nm)^[c]^	r_0_ (mmol/h)^[d]^	Accessiblemol Ru^[e]^ (CO)	TOF (h^1^)^[e]^ (CO)	Conv. HMF (%)	Select. DFF (%)
1	0.5 % Ru/C	0.58	0.89	0.60	5.7E‐06	105	80	100
2	1 % Ru/C	1	1.4	0.78	6.0E‐06	130	87	100
3	3 % Ru/C	3	1.6	0.93	6.1E‐06	153	99	100
4	6 % Ru/C	6	1.8	0.37	6.2E‐06	60	88	100
5	10 % Ru/C	9.8	2	0.25	5.2E‐06	51	85	100
6^[f]^	C	–	–	0.16	–	–	20	100

[a] Reaction conditions: HMF (0.5 mmol), HMF/Ru molar ratio=84, 6 bar O_2_, 120 °C, 4 mL TFT, 1000 rpm, 6 h. [b] Determined by FRX (fluorescence). [c] Particle size determined by STEM. [d] Initial reaction rate has been calculated using conversions below 20 %. [e] Accessible moles of Ru and TOF are calculated with data of CO chemisorption analysis. [f] Blank of reaction, 20 mg of C was used.

### Stability and Reusability of the 3 %Ru/C Catalyst

**Figure 3:**
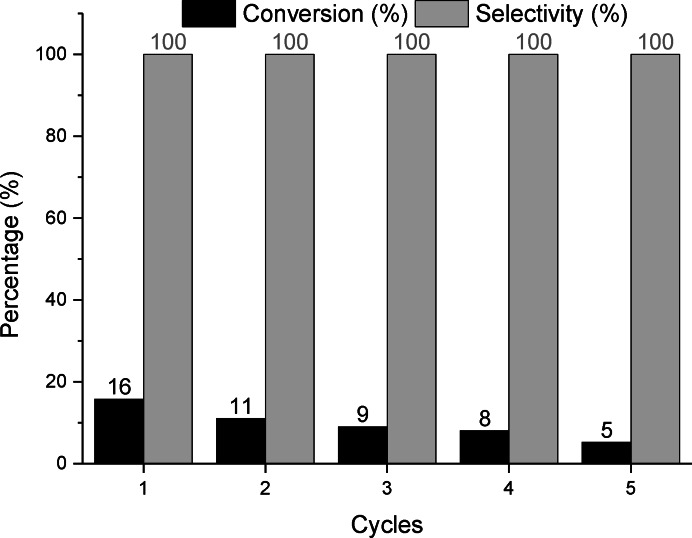
Stability tests of 3 % Ru/C in the oxidation of HMF. Reaction conditions: HMF (0.5 mmol), 20 mg Ru/C catalyst, TFT (4 mL), 120 °C and 6 bar of O_2_ at 5 min reaction time.

The stability and reusability of the optimum 3 %Ru/C catalyst was investigated in the oxidation reaction under a pressure of 6 bars of oxygen at 120 °C and working at lower level of conversion (less than 20 %). After the reaction, the catalyst was recovered by filtration and reused in the next cycle. As can be seen in Figure 3, the 3 % Ru/C catalyst was reused five consecutive cycles and a gradual decrease of conversion was observed along the different reuses, although the selectivity to DFF was maintained at 100 % (kinetics are presented in Figure S3).

In addition, to verify if the catalytic process involved Ru in solution, an additional experiment was carried out where the oxidation of the HMF with 3 % Ru/C was stopped after 2 h of reaction. At this point, the catalyst was filtered in hot, and the reaction continued until 24 h, with no conversion observed (Figure S4). Furthermore, the inductively coupled plasma atomic spectroscopy (ICP‐AES) analysis of the reaction crude showed the complete absence of Ru, indicating that there was not Ru leaching during the reaction (Table S2). The analysis of the used catalyst by transmission scanning microscope (STEM) (see Figure S5) showed that the particle size increases from 1.6 nm of the fresh catalyst to 2 nm, which would explain the differences in performance during the reuses, and the loss of conversion from 99 to 85 % of DFF yield.

### Oxidation Reaction of 5‐HMF to DFF using Ru/C Doped with K, Cs and Na

Considering the results presented above, and in order to study the effect of the presence of alkali promoters on the activity and stability of the catalyst, we prepared tree samples of the 3 %Ru/C bearing Na, K, Cs, as promoters. The promoters were introduced into the catalyst by impregnation of the carbon support with aqueous solutions of their salts (nitrates) containing 450 ppm of the alkali, followed by the impregnation with the salt precursor of Ru (RuCl_3_ ⋅ 3H_2_O). The catalytic study of the oxidation reaction of HMF (see Table 3 and Figure S6) showed that in general, the initial rates of all promoted catalyst are higher than the unpromoted sample (entry 4), being the catalyst promoted with potassium the most active, with an order of activity 3 %Ru/C‐K_450_>3 %Ru/C‐Na_450_>3 %>Ru/C–Cs_450_ Furthermore, the Ru/C−K catalyst resulted the most selective (100 % of DFF yield at 3 h of reaction), meanwhile the Ru/C−Na and Ru/C−Cs samples were less selective since lower amounts of overoxidation products were detected.

**Table 3:** Oxidation of 5‐hydroxymethylfurfural with 3 % Ru/C catalysts doped with alkali metals.^[a]^

Entry	Catalyst	Alkali (ppm) ^[b]^	r_0_ (mmol/h) ^[c]^	Acc.mol Ru^[d]^	TOF (h^−1^)^[d]^	Time (h)	Conv. HMF (%)	Yield DFF (%)	Yield FFCA (%)	Select.DFF (%)
1	3 % Ru/C‐Cs_450_	423.5	0.99	3.87E‐6	256	3	71	70	1	99
						6	95	90	5	95
2	3 % Ru/C‐Na_450_	443.7	1.05	3.8E‐6	275	3	89	88	1	99
						6	99	95	4	96
3	3 % Ru/C‐K_450_	453.2	1.4	4E‐6	350	3	100	100	–	100
4	3 % Ru/C	–	0.93	6.1E‐6	153	6	99	99	–	100

[a] Reaction conditions: HMF (0.5 mmol), HMF/Ru molar ratio=84, 6 bar O_2_, 120 °C, 4 mL TFT, 1000 rpm. [b] Determined by ICP. [c] Initial speed has been calculated using conversions below 20 %. [d] Accessible moles of Ru and TOF are calculated with data of CO chemisorption analysis.

Hence, we synthesize Ru/C catalysts dopped with K loadings ranging from 100 up to 900 ppm of K, and were screened under the same reaction conditions at 120 °C, 6 bar O_2_ for 5 h (see Table 4, Figure S7). STEM analysis show the fresh 3 % Ru/C‐K_450_ catalyst present the same particle size than the undoped sample (1.6 nm).

**Table 4:** Oxidation of 5‐hydroxymethylfurfural with different loadings of potassium in 3 % Ru/C catalysts.^[a]^

Entry	Catalyst	r_0_ (mmol/h)	Conv. HMF (%)	Yield DFF (%)	Select. DFF (%)
1	3 % Ru/C‐K_100_	0.35	89	89	100
2	3 % Ru/C‐K_260_	0.76	93	93	100
3	3 % Ru/C‐K_450_	1.4	100	100	100
4	3 % Ru/C‐K_580_	0.94	93	93	100
5	3 % Ru/C‐K_900_	0.78	92	92	100

[a] Reaction conditions: HMF (0.5 mmol), HMF/Ru=84 (20 mg of catalyst), 6 bar O_2_, 120 °C, 1000 rpm, 5 h.

According to the results summarized in Table 4, 3 % Ru/C‐K_450_ display the highest efficiency for the HMF oxidation reaction, with the highest activity and yield at 5 h.

**Figure 4:**
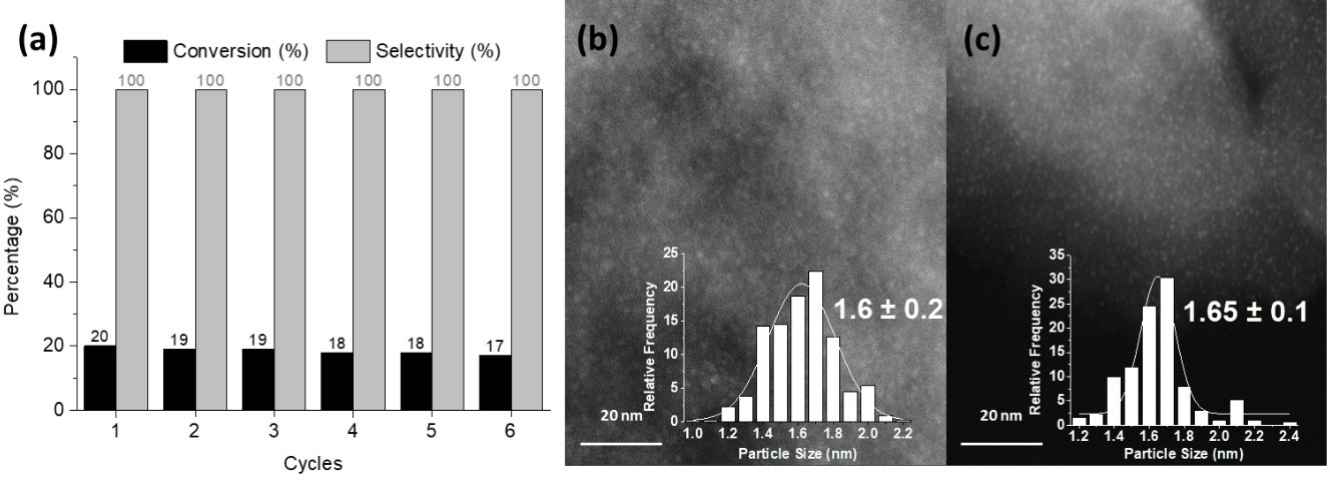
(a) Stability tests of 3 % Ru/C‐K_450_ in the oxidation of HMF. Reaction conditions: HMF (0.5 mmol), 20 mg Ru/C catalyst, TFT (4 mL), 120 °C and 6 bar of O_2_ at 5 min reaction time. (b) STEM image of the fresh 3 % Ru/C‐K_450_ catalyst. (c) STEM image of the used 3 %Ru/C‐K_450_ catalyst after six cycles.

Finally, we have studied the catalyst recyclability for the reaction using 3 % Ru/C‐K_450_. As shown in Figure 4a (see kinetics in Figure S8), this catalyst was reused for six consecutive runs maintaining activity and selectivity. No leaching of ruthenium or potassium occurred in the reaction medium, as revealed by inductively coupled plasma‐mass spectrometry (ICP) analysis of the filtrates. TEM analysis of the recycled catalyst showed that, as in the fresh catalyst, it is formed by monodispersed metal NPs (Figures 4b and c). While the metal size distribution obtained by STEM analysis showed a partial sinterization of the smaller nanoparticles for the used catalyst, the average metal crystal size was maintained around 1.6 nm. These results indicate that the presence of the K promoter improves the stability to the NPs, decreasing the rate of metal agglomeration, allowing a prolonged use.

### Catalytic Active Sites and Reaction Mechanism

In order to explain the above reported catalytic data, spectroscopic characterization have been carried out on selected samples. On one hand, the effect of the promoter on the electronic properties of ruthenium metal species was analysed by UV‐VIS spectroscopy employing tetracyanoethylene (TCNE) as a single‐electron acceptor. TCNE has four cyano groups connected to the central C=C double bond with low energy π* orbitals. When an electron is added to TCNE, it formed a blue‐coloured electron‐TCNE complex (TCNEC^**−**^), which give a characteristic peak at around 300 nm, indicating the formation of the anion radical, providing evidence of an electron‐transfer reaction.^[45]^ Using this methodology and as shown in Figure S9‐a, the intensity of the adsorption peak at 300 nm is maxima in the 3 % Ru/C−K sample, following the trend 3 % Ru/C−K>3 % Ru/C‐Na>>3 % Ru/C−Cs. Interestingly, similar trend is observed in the catalytic performance displayed in Table 3, underlining the effect of the Ru electronic properties on the catalytic performance. In addition to the UV‐VIS study, the presence of electron rich ruthenium species in the 3 % Ru/C‐K_450_ sample is confirmed by X‐ray photoelectron spectroscopy (XPS) (details in SI, Figure S11 and Table S3). In particular, a shift in the Ru3d_5/2_ core line to lower binding energy (280.0 eV) is observed in the Ru^0^ component in the presence of K compared to that of the un‐promoted sample (280.6 eV). Furthermore, in the K promoted sample the concentration of surface oxygen species determined by XPS is markedly lower than in the 3 % Ru/C–Cs (Table S4). A high oxygen concentration may indicate the prevalence of alkali oxide domains, while low concentration of surface oxygen species suggests highly dispersed alkali ions with low tendency to form oxide like agglomerate, thus interacting more favourable with the Ru metal and resulting in a more effective electron transfer from the dopant to the metal site. Hence, from the aforementioned results we can conclude in an enhanced interaction of ruthenium species with K, endorsed by its higher dispersion and boosting the stabilization of electron rich Ru species (Ru^δ−^). In addition to these results where the nature of the alkali ion influences the catalytic performance, the loading of the promoter also plays a role. In this direction, based on UV‐VIS studies, a good correlation between the electronic properties of the ruthenium species and the catalytic properties is also observed at different loadings of the promoter. Thus, from the catalytic data displayed in Table 4, the promoting effect of K is maxima at 450 ppm of K, matching perfectly the trend observed in the UV‐VIS spectra of adsorbed TCNE, where the intensity of the 300 nm band is maxima at the same level of the promoter (Figure S10).

**Figure 5:**
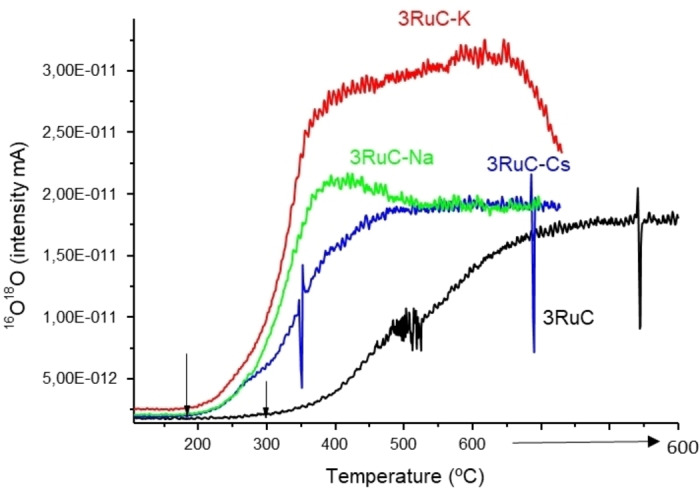
16O_2_–18O_2_ isotopic studies for 3 % Ru/C and 3 % Ru/C‐K, 3 % Ru/C–Cs and 3 % Ru/C‐Na samples.

In order to get deeper insights into the reaction mechanism, operando Raman studies and 16O_2_–18O_2_ isotopic studies are done. Previous studies done in our group showed that the activation of molecular oxygen and the nature of active oxygen species may dependent on the electronic properties of the metal site, influencing accordingly the catalytic performance.[^46^, ^47^] In those studies, a different activity has been observed between dissociated atomic oxygen species and lattice oxygen species coming from surface oxidation of the metal particle. In the particular case of the HMF oxidation over a Ru/C catalyst, Nie et al.,^[26]^ concluded in a reaction mechanism involving dissociative oxygen activation with the stabilization of atomic oxygen species on the catalyst surface. In this type of mechanism, electron rich ruthenium species are expected to favour O_2_ activation in a process involving back donation from the metal d band to the π* anti orbital of O_2_, weakening the O−O bond and favouring O_2_ activation. Indeed, this is exactly what is observed in the 16O_2_–18O_2_ isotopic studies of Figure 5, where a markedly lower oxygen activation ability is observed on the un‐promoted (i. e., 3 % Ru/C) sample (black line), with an onset temperature of ∼306 °C, compared to the promoted ones where the onset temperature is markedly lower around 197 °C).

Moreover, in the promoted samples, the oxygen exchange capacity increase in the order 3 % Ru/C−Cs ≤ 3 % Ru/C‐Na <3 % Ru/C−K, following similar trend as observed from the electronic state of the Ru species determined by UV‐VIS of TCNE. Thus, we can say that electron rich Ru species favour molecular oxygen activation (3 % Ru/C−K) whereas it is less favoured on electro deficient Ru species (3 % Ru/C).

Interestingly, from operando Raman studies, we found that not only the activation of molecular oxygen is influenced by the electronic properties of the ruthenium species, but also the nature of the dissociated oxygen species. In fact, Raman studies in the presence of O_2_ at increasing temperatures from 60 to 120 °C done on the 3 % Ru/C sample shows the formation of ruthenium oxide surface species with characteristic bands, (namely E_g_, A_1g_ and B_2g_) located at 537, 635 and 717 cm^−1^ together with 155 and 212 cm^−1[48]^ (Figure S12‐a), whereas this behaviour is not observed on the most electrophilic 3 % Ru/C‐K_450_. In this last case a weak band at 448 cm^−1^ is observed at 60 °C, while disappear at higher temperature (120 °C) (Figure S12‐b). Similar band, but less intense, is also observed on the pure carbon support when exposed to O_2_ conditions, but at 120 °C (Figure S12‐c). Notice that pure carbon shows some activity in the oxidation of HMF as displayed in Table 2, entry 6. Accordingly, in the 3 % Ru/C‐K_450_ sample, we may propose the existence of weakly interacting oxygen atoms coming from the dissociation of molecular oxygen on the Ru NP which can migrate to the carbon. Furthermore, similar to the 3 % Ru/C sample, ruthenium oxide formation is also detected but in a lower extension on the Na (3 % Ru/C−Na) promoted sample. These differences among the samples can be explained by the different electron properties of the Ru species. Hence, electron deficient ruthenium species (as in the 3 % Ru/C sample) exhibits a higher susceptibility toward oxidation under working conditions due to its more oxophilic nature, whereas electron rich ruthenium species (as in 3 % Ru/C−K) favours O_2_ activation while preventing bulk metal oxidation. This oxygen species has a weak interaction with the Ru surface, facilitating spillover over the C support. In the work of Liu et al.,^[26]^ adsorbed atomic oxygen species, derived from O_2_ dissociation on the Ru surface, have been proposed as active species, resulting in high catalytic activity. Accordingly, in our work, the higher activity observed on the 3 % Ru/C−K sample versus the 3 % Ru/C and the 3 % Ru/C−Na samples, can be ascribed to the different nature of oxygen species, i. e., atomic oxygen species in the first one whereas lattice oxygen due to surface oxidation of the ruthenium nanoparticle in the 3 % Ru/C and 3 % Ru/C−Na samples.

**Figure 6:**
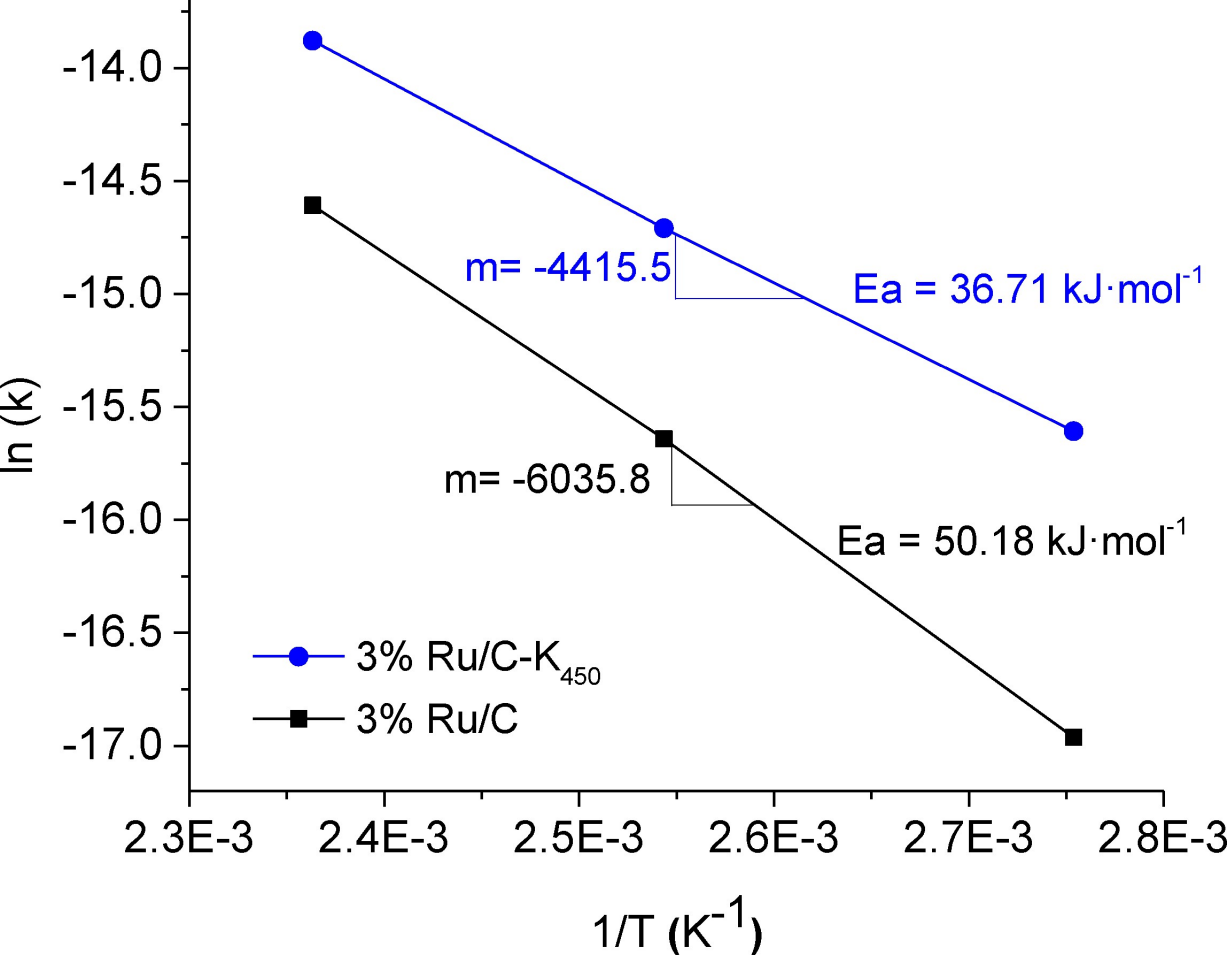
Activation energies of 3 % Ru/C and 3 % Ru/C‐K_450_ samples.

This result agrees with the different apparent activation energy to DFF observed in the 3 % Ru/C and 3 % Ru/C‐K_450_ samples (Figure 6), which may indicate different reaction mechanisms or involvement of different active species in both samples.

In conclusion, we have shown the possibility to modulate the catalytic activity of Ru/C catalysts by tuning the electronic properties of the ruthenium nanoparticle. In definitive we can use the electron properties of the ruthenium species, as a good descriptor of the catalytic activity in the oxidation of HMF to DFF using molecular oxygen.

### Scope of the Reaction

To expand the scope of the aerobic oxidation of alcohols using the 3 % Ru/C‐K_450_ catalyst, the oxidation of various alcohols has been performed and the results are presented in Table 5. As can be seen, benzyl alcohols carrying both electron‐donating and electron‐withdrawing groups give the corresponding benzaldehyde with high yields and selectivity. However, benzyl alcohols with electron‐withdrawing groups display decreased reactivity and require longer reaction times, as describe other authors.^[49]^ Aerobic oxidation of cinnamyl alcohol and 1‐phenyl ethanol resulted in high yield (82 and 80 %, respectively). The oxidation of 1‐octanol gives moderated yield of octanal (50 %) owing to the low reactivity of primary aliphatic alcohols.

**Table 5:** Oxidation of different alcohols using 3 % Ru/C‐K_450_ as catalyst.^[a]^

Substrate	Product	Time (h)^[c]^	YieldAldehyde (%)^[b]^	Selectivity (%)
Benzyl alcohol	Benzyl aldehyde	2	96	96
4‐Methoxybenzyl alcohol	4‐Methoxybenzyl aldehyde	1.5	95	100
4‐Chlorobenzyl alcohol	4‐Chlorobenzyl aldehyde	2	98	100
Cinnamyl alcohol	Cinnamyl aldehyde	2	82	100
1‐Phenylethan‐1‐ol	Acetophenone	6	80	100
Octanol	Octanal	8	50	100
Piperonyl alcohol	Piperonal	1.5	100	100

[a] Reaction conditions: alcohol (0.5 mmol), 3 % Ru/C‐K_450_ (20 mg), trifluorotoluene (4 mL), at 120 °C under 6 bar pressure of oxygen. [b] Determined by GC. [c] Time at which the maximum yield of the aldehyde is obtained.

From the results presented above, we can conclude that our catalytic system exhibits high activity in the aerobic oxidation of a variety of alcohols.

## Experimental Section

### Catalysts Preparation

All the reagents used were of analytical quality. The following reagents were purchased from Sigma‐Aldrich: 5‐hydroxymethylfurfural (≥99 %), Diformylfuran (≥98 %), n‐hexadecane (≥99 %), and 5 % ruthenium on carbon. 2,5‐Furandicarboxylic acid (FDCA) was provided by Apollo Scientific. 5‐Hydroxymethyl‐2 furancarboxylic acid (HMFCA) was purchased from Carbosynth. The following reagents were supplied by ABCR: ruthenium (III) chloride trihydrate (95 %) and trifluorotoluene. Potassium nitrate was provided by Panreac Reagent and 1 % ruthenium on carbon was obtained from Johnson Mattehey. The Carbon NORIT® RX 3 extra was supplied by NORIT company.

The carbon black, RX3 Extra (Norit) was used as support. The different loading catalysts (0.5, 1.5, 3, 5, 6 and 10 % Ru/C) were prepared through the deposition‐precipitation method, impregnating the appropriate amount of carbon (500 mg) with a pure solution of ruthenium chloride trihydrate in 2 mL of acetone. After stirring overnight at room temperature, the solvent was filtered, and the catalyst was washed with copious amounts of milli‐Q water to remove the chlorides present. The catalyst was dried in an oven at 80 °C. Then, it was reduced with a stream of H_2_ (50 mL min^−1^) at 400 °C for 4 h with a ramp of 10 °C/min to clean the catalyst from chloride and other carbon deposits, and passivated.

The promoters (K, Cs, Na) were introduced by impregnation from aqueous solutions of their respective nitrate salts (15, 20, 35, 45 and 60 wt %). Only a few parts per million remain impregnated in the carbon, as shows ICP results in Table S2. First, carbon was performed by the impregnation from the aqueous solution of salt, followed by drying in air. After stirring overnight at room temperature, the solution was filtered and the solid obtained was washed with abundant milli‐Q water to remove traces of nitrate. Subsequently, the ruthenium support was carried out in a similar process to that mentioned above.

### Experimental Catalytic Activity

The catalytic experiments were carried out in a 12.5 mL capacity stainless‐steel high‐pressure reactor. To a solution of HMF (0.5 mmol) in 4 mL of solvent was added the catalyst. After sealing the reactor, the air was removed by two consecutive purges with 5 bar N_2_. Subsequently, the mixture of substrates and catalyst was kept under magnetic stirring, heated to the desired temperature, and pressurized with 6 bars of O_2_ (t=0 of the reaction). All experiments were carried out at 1000 rpm.

During the experiment, the pressure was kept constant, and aliquots were taken from the reactor (approximately 50 μL) at different reaction times. The products were analysed on a high‐performance liquid chromatograph (HPLC) (Agilent 1260 Infinity II LC System), an IR detector and an Aminex HPX‐87H column (Biorad). The mobile phase is a filtered acidified water solution (0.005 M H_2_SO_4_ mobile phase, 0.6 mL/min flow). 1,3‐propanediol was used as an external standard. Additionally, the evolution of the reactant and product with time was also analyzed using GC (HP5 column) (see Figure S13) and ^1^H‐NMR spectroscopy (Figure S14). The identification of the products was carried out by GC‐MS and ^1^H‐NMR. The aliquots were taken during the reaction following this procedure: once the sample was removed from the reactor, the TFT was evaporated and dissolved in another vial where the external standard (6.6 10^−3^ mmol of 1,2‐propanodiol) was added. All samples were then acidified with dilute sulphuric acid to a pH of 1–2. After the reaction, the catalyst was recovered by filtration and subjected to Soxhlet extraction using acetone as the washing solvent. Subsequently, the catalyst was activated at 400 °C under a flow of H_2_ (50 mL/min) for 4 hours and reused in the next cycle.

## Conclusions

We have showed that a catalyst based on Ru nanoparticles supported on potassium doped activated carbon is highly active and selective in the anaerobic oxidation of HMF in DFF. Doping with K not only increases the catalytic activity by 2.5 times compared to undoped Ru/C, but also prevents the metal from sintering, while the catalyst is able to maintain its activity after several consecutive reaction cycles. Spectroscopic studies indicate that the higher activity and stability observed in the Ru/C−K catalyst compared to the undoped sample can be mainly ascribed to a different oxidation reaction mechanism, associated with the presence of electron‐enriched Ru species. The electron‐rich Ru species facilitate the dissociation of O_2_ and, at the same time, prevent metal oxidation. These active oxygen species are proposed to be more reactive than lattice oxygen from surface ruthenium oxidation which it is observed to occur in the unpromoted sample. This study demonstrates the possibility of tuning the catalytic activity of Ru/C catalysts for the selective oxidation of HMF to DFF by modifying the electronic properties of ruthenium nanoparticles.

## Conflict of Interests

The authors declare no conflict of interest.

1

## Supporting information

As a service to our authors and readers, this journal provides supporting information supplied by the authors. Such materials are peer reviewed and may be re‐organized for online delivery, but are not copy‐edited or typeset. Technical support issues arising from supporting information (other than missing files) should be addressed to the authors.

Supporting Information

## Data Availability

The data that support the findings of this study are available in the supplementary material of this article.

## References

[1] F. Martins , C. Felgueiras , M. Smitkova , N. Caetano , Energies (Basel) 2019, 12, 946, DOI 10.3390/en12060964.

[2] E. Palm , J. Hasselbalch , K. Holmberg , T. D. Nielsen , Env. Polit. 2022, 31, 365–385.

[3] A. Corma Canos , S. Iborra , A. Velty , Chem. Rev. 2007, 107, 2411–2502. 17535020 10.1021/cr050989d

[4] T. Ståhlberg , W. Fu , J. M. Woodley , A. Riisager , ChemSusChem 2011, 4, 451–458. 21275065 10.1002/cssc.201000374

[5] A. A. Rosatella , S. P. Simeonov , R. F. M. Frade , C. A. M. Afonso , Green Chem. 2011, 13, 754–793.

[6] S. P. Teong , G. Yi , Y. Zhang , Green Chem. 2014, 16, 2015–2026.

[7] M. E. Zakrzewska , E. Bogel-Łukasik , R. Bogel-Łukasik , Chem. Rev. 2011, 111, 397–417, DOI 10.1021/cr100171a. 20973468

[8] J. J. Bozell , G. R. Petersen , Green Chem. 2010, 12, 539–55.

[9] O. Casanova , S. Iborra , A. Corma , ChemSusChem 2009, 2, 1138–1144, DOI 10.1002/cssc.200900137. 19760702

[10] B. Saha , S. Dutta , M. M. Abu-Omar , Catal. Sci. Technol. 2012, 2, 79–81, DOI 10.1039/c1cy00321f.

[11] M. El Fergani , N. Candu , P. Granger , S. M. Coman , V. I. Parvulescu , Catal. Today 2022, 405–406, 267–276, DOI 10.1016/j.cattod.2022.04.033.

[12] S. Zhang , Z. Chen , J. F. Gu , W. Sang , M. Jiang , S. Li , P. Wang , Z. Kou , C. Chen , Chem. Rec. 2023, 23, e202300019, DOI 10.1002/tcr.202300019. 37017486

[13] J. Ma , Z. Du , J. Xu , Q. Chu , Y. Pang , ChemSusChem 2011, 4, 51–54. 21226210 10.1002/cssc.201000273

[14] A. S. Amarasekara , D. Green , L. T. D. Williams , Eur. Polym. J. 2009, 45, 595–598.

[15] I. Delidovich , P. J. C. Hausoul , L. Deng , R. Pfützenreuter , M. Rose , R. Palkovits , Chem. Rev. 2016, 116, 1540–1599. 26523853 10.1021/acs.chemrev.5b00354

[16] Q. Girka , N. Hausser , B. Estrine , N. Hoffmann , J. Le Bras , S. Marinković , J. Muzart , Green Chem. 2017, 19, 4074–4079.

[17] K. I. Galkin , F. A. Kucherov , O. N. Markov , K. S. Egorova , A. V. Posvyatenko , V. P. Ananikov , Molecules 2017, 22, 2210, DOI 10.3390/molecules22122210. 29231880 PMC6149738

[18] H. Xia , S. Xu , H. Hu , J. An , C. Li , RSC Adv. 2018, 8, 30875–30886. 35548764 10.1039/c8ra05308a PMC9085621

[19] Q. Ke , Y. Jin , F. Ruan , M. N. Ha , D. Li , Y. Cao , H. Wang , T. Wang , V. N. Nguyen , X. Han , X. Wang , P. Cui , P. Cui , Green Chem. 2019, 21, 4313–4318, DOI 10.1039/c9gc01041f.

[20] P. Pal , S. Saravanamurugan , ChemCatChem 2020, 12, 2324–2332, DOI 10.1002/cctc.202000086.

[21] Y. Zhang , W. Li , Y. Cao , M. Chen , W. Li , J. Zai , A. Iqbal , R. Qi , X. Qian , ChemSusChem 2022, 15, e202102596, DOI 10.1002/cssc.202102596. 34927792

[22] Y. Zhang , D. Ma , Z. Chen , A. Iqbal , J. Hu , M. Chen , X. Liu , T. T. Tsega , H. Fazal , P. Xu , F. Tao , J. Zai , X. Qian , ACS Sustainable Chem. Eng. 2022, 10, 541–551, DOI 10.1021/acssuschemeng.1c07037.

[23] P. Hoang Tran , ChemSusChem 2022, 15, e202200220, DOI 10.1002/cssc.202200220. 35307983

[24] W. Zhang , H. Qian , Q. Hou , M. Ju , Green Chem. 2023, 25, 893–914.

[25] S. Biswas , A. Pal , T. Pal , RSC Adv. 2020, 10, 35449–35472. 35515660 10.1039/d0ra06168a PMC9056907

[26] J. Nie , J. Xie , H. Liu , J. Catal. 2013, 301, 83–91.

[27] T. E. Hoost , J. G. Goodwin , J. Catal. 1991, 130, 283–292.

[28] W. D. Mross , Catal. Rev. 1983, 25, 591–637.

[29] J. Yu , K. Wang , S. Shao , W. Li , S. Du , X. Chen , C. Chao , X. Fan , Chem. Eng. J. 2023, 458, 141486, DOI 10.1016/j.cej.2023.141486.

[30] E. Truszkiewicz , W. Raróg-Pilecka , M. Zybert , M. Wasilewska-Stefańska , E. Topolska , K. Michalska , Pol. J. Chem. Technol. 2014, 16, 106–110, DOI 10.2478/pjct-2014-0079.

[31] W. Raróg-Pilecka , D. Szmigiel , Z. Kowalczyk , S. Jodzis , J. Zielinski , J. Catal. 2003, 218, 465–469.

[32] H. S. Zeng , K. Inazu , K. Aika , J. Catal. 2002, 211, 33–41.

[33] I. Rossetti , N. Pernicone , L. Forni , Appl. Catal. A 2001, 208, 271–278.

[34] Z. Kowalczyk , S. Jodzis , W. Raróg , J. Zieliński , J. Pielaszek , Appl. Catal. A 1998, 173, 153–160.

[35] W. Raróg , Z. Kowalczyk , J. Sentek , D. Składanowski , J. Zieliński , Catal. Lett. 2000, 68, 163–168.

[36] B. Lin , K. Wei , X. Ma , J. Lin , J. Ni , Catal. Sci. Technol. 2013, 3, 1367–1374.

[37] S. Adjimi , J. M. García-Vargas , J. A. Díaz , L. Retailleau , S. Gil , M. Pera-Titus , Y. Guo , A. Giroir-Fendler , Appl. Catal. B 2017, 219, 459–466.

[38] S. Cao , J. R. Monnier , C. T. Williams , W. Diao , J. R. Regalbuto , J. Catal. 2015, 326, 69–81.

[39] S. T. Hussain , J. Trace Microprobe Tech. 1996, 14, 681–693.

[40] K. ichi Aika , Catal. Today 2017, 286, 14–20.

[41] A. Ozaki , K. Aika , H. Hori , Bull. Chem. Soc. Jpn. 1971, 44, 3216–3216.

[42] K. ichi Aika , H. Hori , A. Ozaki , J. Catal. 1972, 27, 424–431.

[43] M. Boudart , Chem. Rev. 1995, 95, 661–666, DOI 10.1021/cr00035a009.

[44] C. J. H. Jacobsen , S. Dahl , P. L. Hansen , E. Törnqvist , L. Jensen , H. Topsøe , D. V. Prip , P. B. Møenshaug , I. Chorkendorff , J. Mol. Catal. A 2000, 163, 19–26.

[45] F. Wang , W. Ueda , J. Xu , Angew. Chem. Int. Ed. 2012, 51, 3883–3887. 10.1002/anie.201105922 22415843

[46] P. Concepción , M. Boronat , S. García-García , E. Fernández , A. Corma , ACS Catal. 2017, 7, 3560–3568.

[47] L. Liu , T. Matsushita , P. Concepción , A. Leyva-Pérez , A. Corma , ACS Catal. 2016, 6, 2211–2221.

[48] S. Bhaskar , P. S. Dobal , S. B. Majumder , R. S. Katiyar , J. Appl. Phys. 2001, 89, 2987–2992.

[49] K. Yamaguchi , N. Mizuno , Chem. Eur. J. 2003, 9, 4353–4361, DOI 10.1002/chem.200304916. 14502621

