# Supplementary material for: Oxidation of 5‐Hydroxymethylfurfural into 2,5‐Diformylfuran on Alkali Doped Ru/C Catalysts. Electron Properties of Ruthenium Species as Descriptor of Catalytic Activity
Source: ChemSusChem. 2024 Oct 25;18(2):e202401515. doi: 10.1002/cssc.202401515 (PMC11739825; doi:10.1002/cssc.202401515)
Supplement: Supplementary file 1 — Supporting Information [file CSSC-18-e202401515-s001.pdf]

ChemSusChem

Supporting Information

Oxidation of 5-Hydroxymethylfurfural into 2,5-Diformylfuran on Alkali Doped Ru/C Catalysts. Electron Properties of Ruthenium Species as Descriptor of Catalytic Activity

Beatriz Hurtado, Karen S. Arias, Patricia Concepción, Maria J. Climent, Sara Iborra,* and Avelino Corma*

Oxidation of 5-Hydroxymethylfurfural into 2,5-Diformylfuran on Alkali Doped Ru/C Catalysts. Electron Properties of Ruthenium Species as Descriptor of Catalytic Activity

SUPPLEMENTARY INFORMATION

Table S1. Summary of heterogeneous ruthenium-supported catalysts reported in the literature, used in the oxidation reaction of HMF to DFF.

**Catalyst** (mg cat.) (%wt Ru)	**HMF (mmol)**	**Ru (mmol)**	**mmol Ru/mmol HMF (%)**	**Solvent (mL)**	**T[a] °C**	**Oxidant (Pressure in bar)**	**Time (h)**	**Conv. HMF (%)**	**Yield DFF (%)**	**TOF (h^{-1})[a]**	**Ref.**
**$Ru(OH)_x/CeO_2$** (25mg) (2.4%Ru)	0.5	5.94E-3	1.2	H_2O (10)	140	O_2 (2.5)	1	75	40	34[b]	[1]
**γ-Fe_2O_3@HAP-Ru** (150mg) Hydroxyapatite (2%Ru)	0.8	0.029	3.6	4-Chlorotoluene (7)	110	O_2 (20mL/min)	2	100	89	4.16[b]	[2]
**Fe_3O_4@SiO_2-NH_2-Ru(III)** (100mg) (14.6%Ru)	0.8	0.144	18	Toluene (7)	120	O_2 (20mL/min)	12	96.1	85.9	1.04[b]	[3]
**$RuCo(OH)_2CeO_2$** (160mg) (6.4%Ru)	1	0.101	10.1	MIBK (7)	120	O_2 (20mL/min)	12	97	83	1.04[b]	[4]
**Ru/CTFs** (50mg) Covalent triazine framworks (4.32%Ru)	1	0.021	2.1	H_2O (15)	140	Air (20)	1	93.7	35.8	-	[5]
**Ru/CTF-a** (58.5mg) (4.32%Ru)	1	0.025	2.5	MTBE (15)	80	Air (20)	3	97	73	-	[6]
**Ru-6C-1N** (45mg) Ru-NP supported on mesoporous lipophilic coreeshell shuttle (1.2%Ru)	1	5.35E-3	0.53	MIBK (30)	105	O2 (1)	8	94	84	13[b]	[7]
**$ZnFe_{1.65}Ru_{0.35}O_4$** (50mg) (12.8%)	0.5	0.063	12.6	DMSO (3)	110	O_2 (20mL/min)	4	100	93.5	2.0	[8]
**Ru-ZrP** (100mg) zirconium phosphate (1.93%Ru)	0.8	0.019	2.4	p-chlorotoluene (7)	110	O_2 (5)	12	100	57	6.9	[9]
**Ru@mPMF** (50mg) poly-melamine-formaldehyde (4.2%Ru)	2	0.021	1.05	Toluene (10)	105	O_2 (20)	12	99.6	85	-	[10]
**$Ru/MnCo_2O_4$** (150mg) (1.8%Ru)	2	0.027	1.35	Toluene (15)	130	O_2 (10)	3	98.3	98.3	63.4[b]	[11]
**Ru_1/NiO** (0.4g)	0.5	0.076	15.2	Toluene	110	O_2	2	91.1	74	57.8	[12]

(1.93%Ru)				(10)		(10)					
**Ru/OMC-$P_{0.56}$** (0.08g) (1%Ru)	2	7.92E-3	0.4	Toluene (25)	90	O_2 (20)	4	100	88	68.7	[13]
**Ru/γ-Al_2O_3** (0.5g) (5%Ru)	4	0.247	6.2	DMF	110	O_2 (5)	3	94.2	79.1	-	[14]
**Ru/HT** (0.1g) (4.41%Ru)	1	0.044	4.4	DMF (3)	120	O_2 (20mL/min)	6	95	92	9.1[b]	[15]
**Ru/γ-Al_2O_3** (200mg) (1.8%Ru)	0.5	0.036	7.2	Toluene (15)	120	O_2 (3)	4	100	97	-	[16]
**Ru/C** (43mg) (3%Ru)	1	0.013	1.3	H_2O (10)	100	O_2 (20)	0.33	91	44.2	51[b]	[17]
**Ru/C** (40mg) (3%Ru)	1	0.012	1.2	Toluene (10)	110	O_2 (20)	0.5	100	96	61.2	[18]
**Ru/C+Pd/C** (21.5mg) (5%Ru)	0.5	0.011	2.4	ACN (2)	110	O_2 (3)	16	100	100	10.4[b]	[19]
**Ru/C** (20mg) (3%Ru)	0.5	5.94E-3	1.2	TFT (4)	120	O_2 (6)	6	99	99	**150**	**This work**
**Ru/C-K_{450}** (20mg) (3%Ru)	0.5	5.94E-3	1.2	TFT (4)	120	O_2 (6)	5	100	100	**240**	**This work**

[a] TOF is expressed as initial reaction rate divided by the total mmol of Ru. [b] TOF has been estimated by the data provided by the authors in the respective reference.

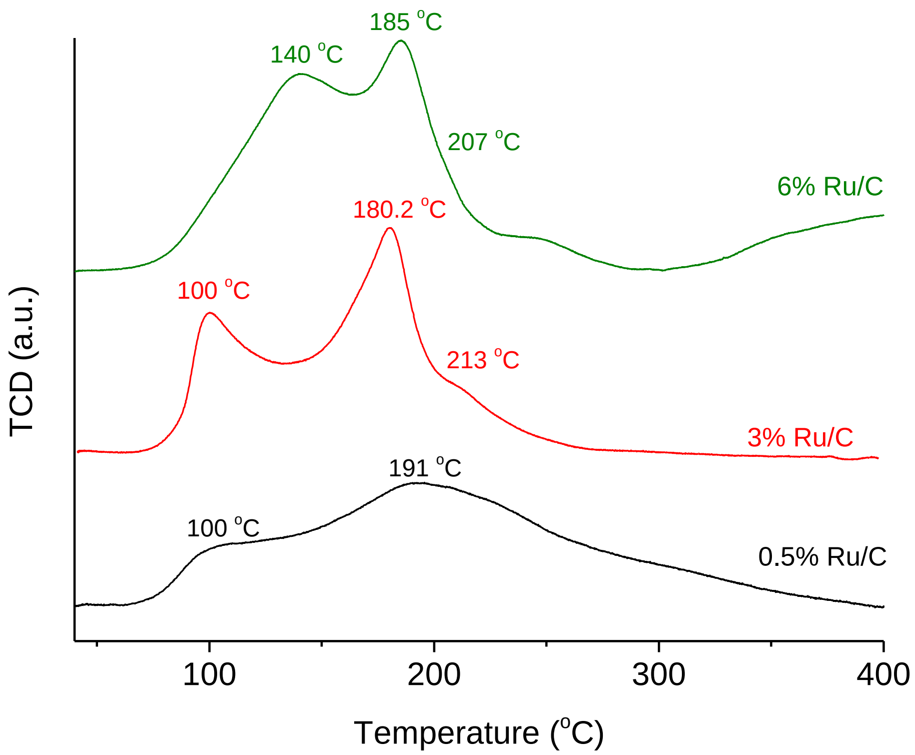

Figure S1. TPR measurements of Ru/C catalysts.

The hydrogen reduction of the samples was studied by H_2-temperature-programmed reduction (H_2-TPR). A mixture of 10 vol.% Hydrogen in Argon (Air Liquide 99.999% purity), total flow of 50 cm^3 min^{-1} (STP), fed a reactor with a ramp of 8 °C min^{-1} from room temperature to 800 °C. 100 mg of catalyst was used in the experiment.

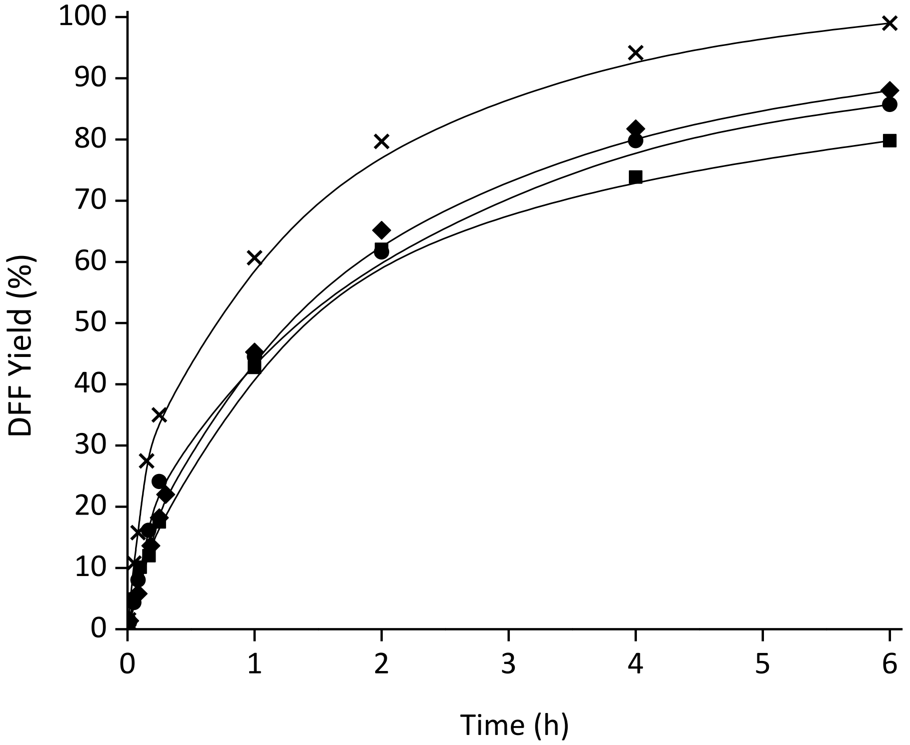

Figure S2. Kinetics of oxidation reaction of HMF to DFF with different loading of Ru on the active carbon. Reaction Conditions: HMF (0.5 mmol), Ru/C catalyst, TFT (4mL), 6 bar O_2, 120 ^oC, 1000 rpm, 6h. 0.5% Ru/C (■), 1% Ru/C (●), 3% Ru/C (×), 6% Ru/C (♦).

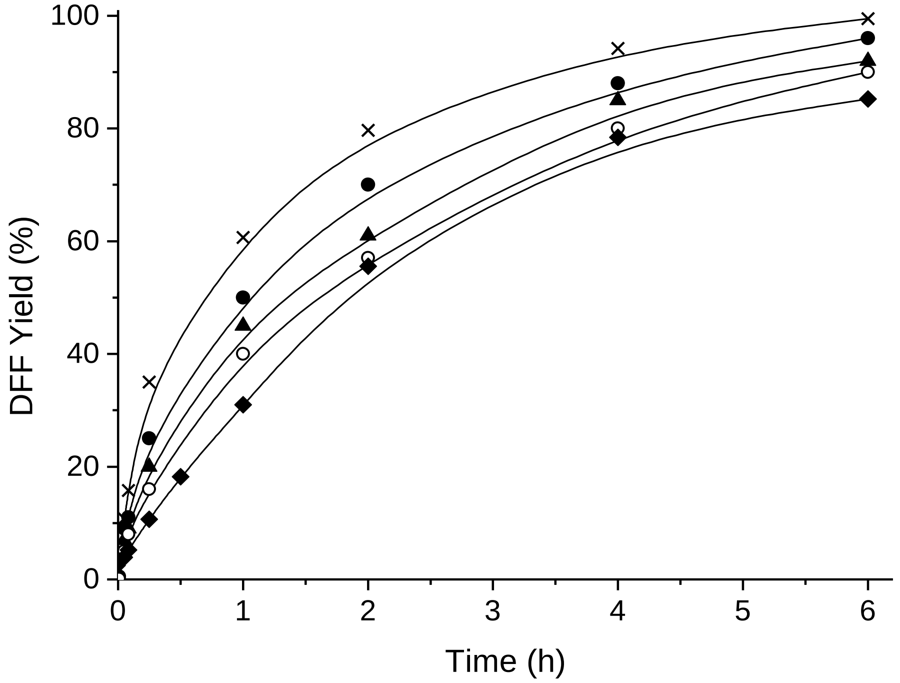

Figure S3. Kinetics of the five consecutive cycles of oxidation of HMF to DFF using 3% Ru/C catalyst. Reaction Conditions: HMF (0.5 mmol), Ru/C catalyst, TFT (4mL), 6 bar O_2, 120 ^oC, 1000 rpm, 6h. 1st cycle (×), 2nd cycle (●), 3rd cycle (▲), 4th cycle (○), 5th cycle (♦).

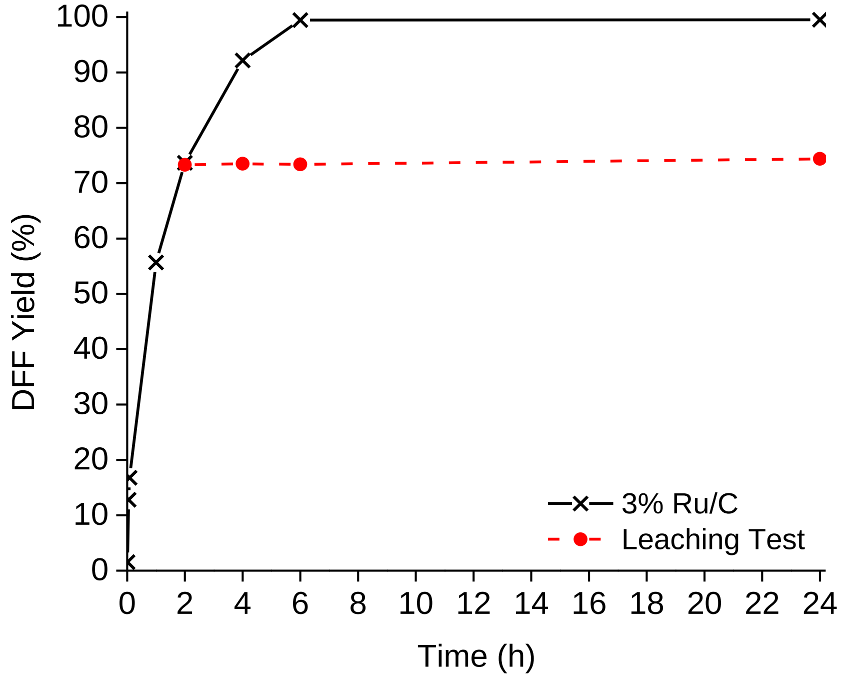

Figure S4. Leaching test of the oxidation reaction of 5-HMF, using 3% Ru/C catalyst.

Reaction conditions: HMF (0.5 mmol), 20 mg Ru/C catalyst, 120 ^{o}C, 6 bar O_2, 24h.

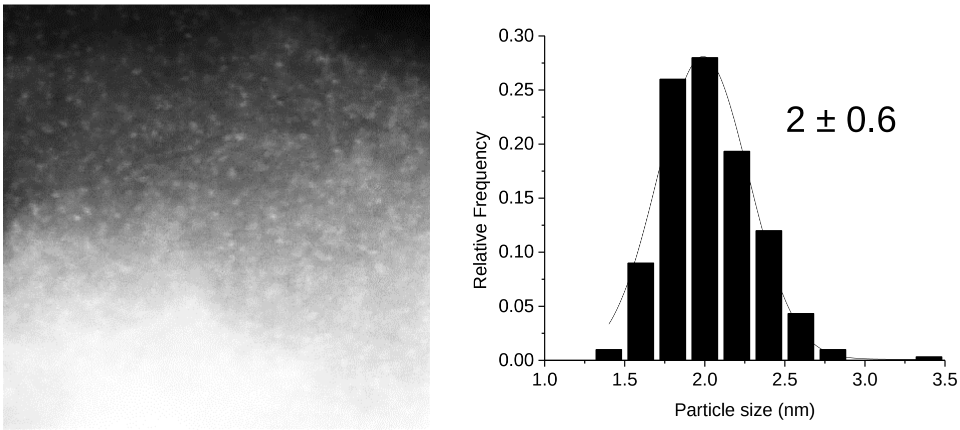

Figure S5. STEM image and particle size distribution of the used 3% Ru/C catalyst after five cycles of reaction. After each reaction, the catalyst was separated from the crude by filtration and thoroughly extracted with acetone in a soxhlet system for 24 hours (<1% of DFF was extracted). The washed catalyst was then dried in an oven at 80 °C for 5h and activated with H_2 at 400 °C (4h).

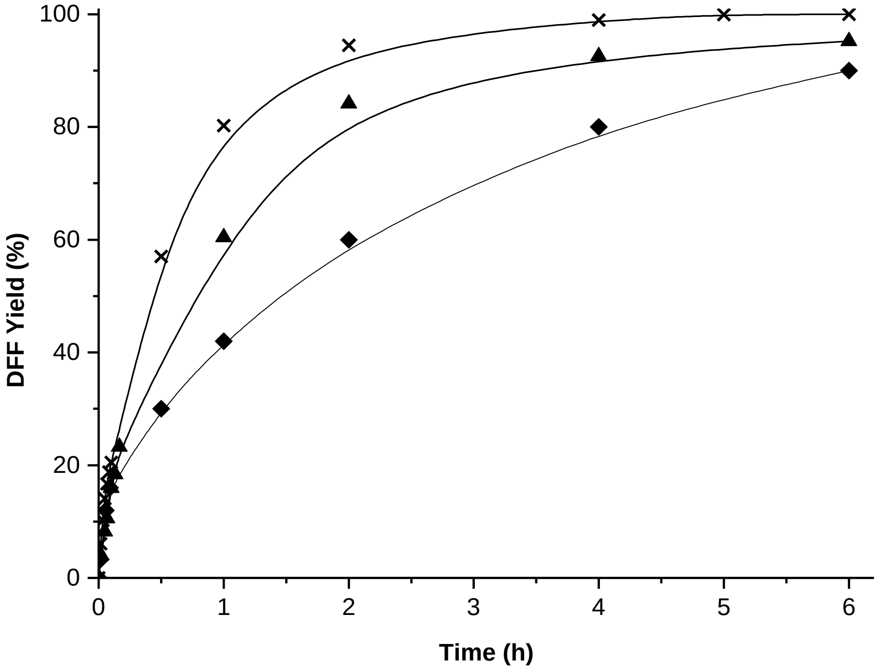

Figure S6. Kinetics of the oxidation of HMF into DFF using 3%Ru/C-M (M: K,Cs,Na) catalyst. Reaction conditions: HMF (0.5 mmol), Ru/C (20 mg), TFT (4 mL), 120 ºC and 6 bar of O_2. 3% Ru/C-K_{450} (×), 3% Ru/C-Cs_{450} (♦), 3% Ru/C-Na_{450} (▲).

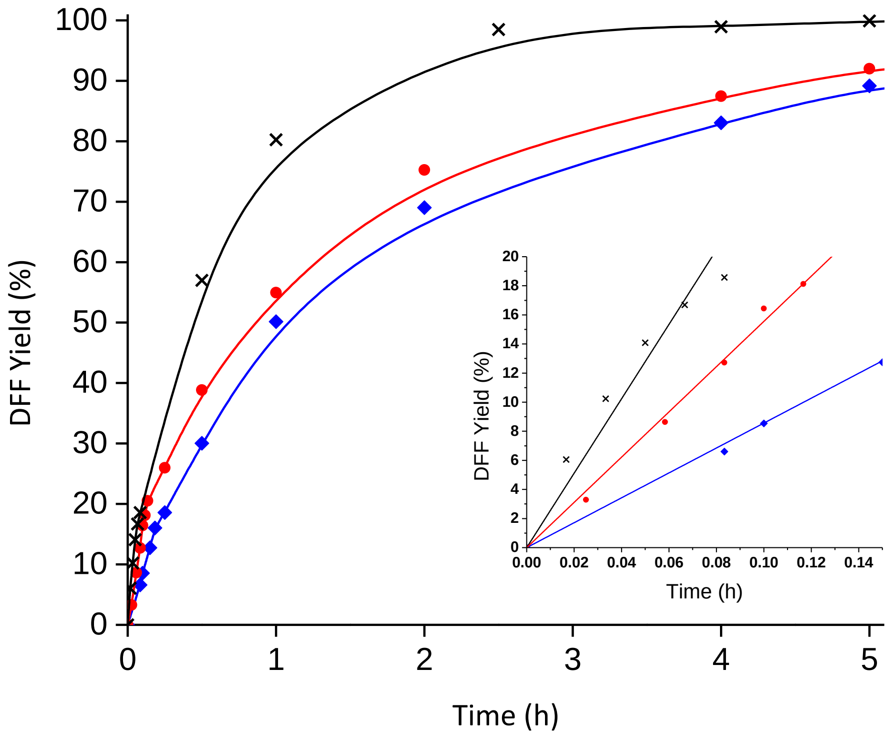

Figure S7. Kinetics of oxidation of HMF to DFF using 3% Ru/C-K catalyst with different K loading. Reaction Conditions: HMF (0.5 mmol), Ru/C-K catalyst (20 mg), TFT (4mL), 6 bar O_2, 120 ^oC, 1000 rpm, 6h. 3% Ru/C-K_{100} (♦), 3% Ru/C-K_{450} (×), 3% Ru/C-K_{900} (●).

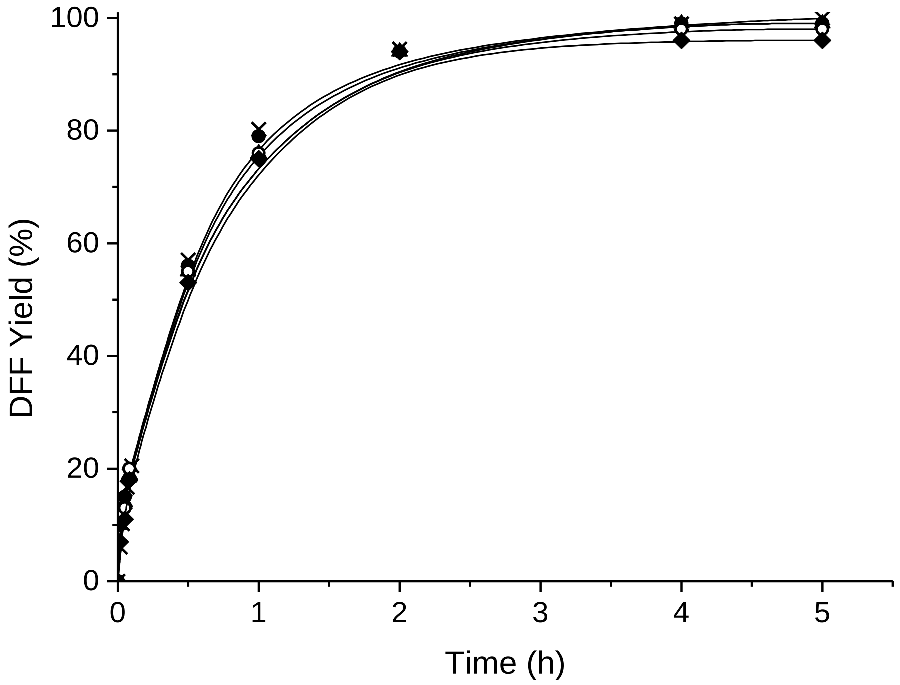

Figure S8. Kinetics of the five consecutive cycles of oxidation reaction of HMF to DFF using 3% Ru/C-K_{450} catalyst. Reaction Conditions: HMF (0.5 mmol), catalyst (20 mg), TFT (4mL), 6 bar O_2, 120 ^oC, 1000 rpm, 5h. 1st cycle (×), 2nd cycle (●), 3rd cycle (▲), 4th cycle (○), 5th cycle (♦).

Table S2. ICP

Sample digestion for performing ICP analysis was performed by the method described below. The weighted material is placed into a 50 mL flask, and 10 mL of H_2SO_4 is added and heated to 90 ^oC, then 10 mL of H_2O_2 (aq.) was added dropwise under stirring. After a few minutes, 2 mL of HNO_3 are added slowly and dropwise and after it is kept under stirring for 2h.

The amount of Ru on carbon support was measured by X-ray fluorescence spectroscopy (XRF), based on a previous calibration by XRF using RuO_2/C mixtures with different known concentrations of Ru. Promoters' content was measured by ICP-OES. According to XRF data, the weight percentage of Ru supported on carbon indicates that the metal is fully supported on the active carbon. However, in the case of K, Na and Cs only a few parts remain supported on carbon.

Catalyst	Ru(wt %)[a]	K (ppm)[b]	Na (ppm)[b]	Cs (ppm)[b]
C Norit RX3	0.0004	0.0002	-	-
3% Ru/C	3	-	-	-
3%Ru/C-K_{100}	3	108	-	-
3%Ru/C-K_{450}	2.95	453.2	-	-
3%Ru/C-K_{900}	3	904.1	-	-
3% Ru/C-Na_{450}	3	-	443.7	-
3% Ru/C-Cs_{450}	3	-	-	423.5

3% Ru/C (5th cycle)	3	-	-	-
3%Ru/C-K_{450} (6th cycle)	2.95	450	-	-

[a] Determined by XRF. [b] Determined by ICP.

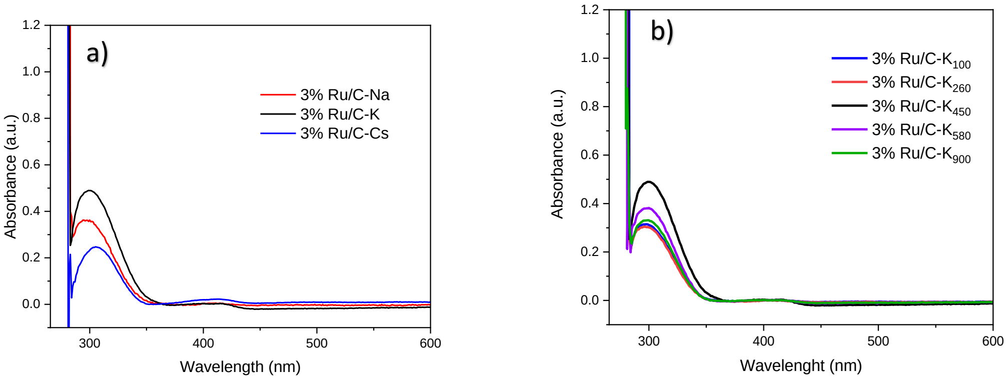

Figure S9. UV-vis absorption spectrum recorded (a) using different promoter metal collected from TCNE/CH_3CN solution in contact with 3% Ru/C-M (K, Na and Cs), and (b) at different percentage of K in contact with 3% Ru/C-K catalysts.

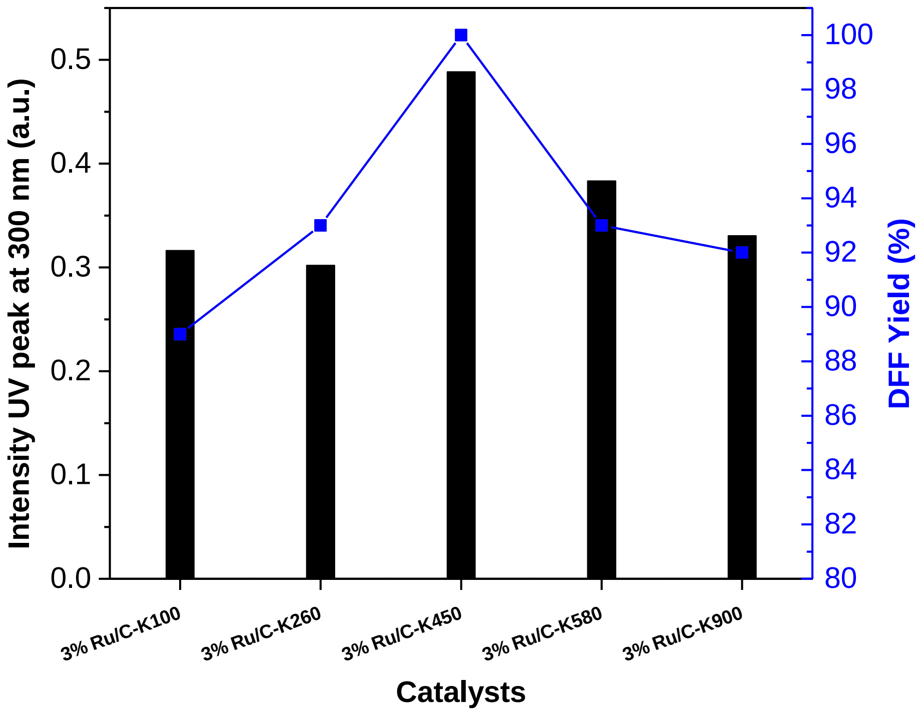

Figure S10. Maxima intensity of the 300 nm band in the UV-VIS spectra of adsorbed TCNE and maxima yield of DFF using different loadings of the promoter (K).

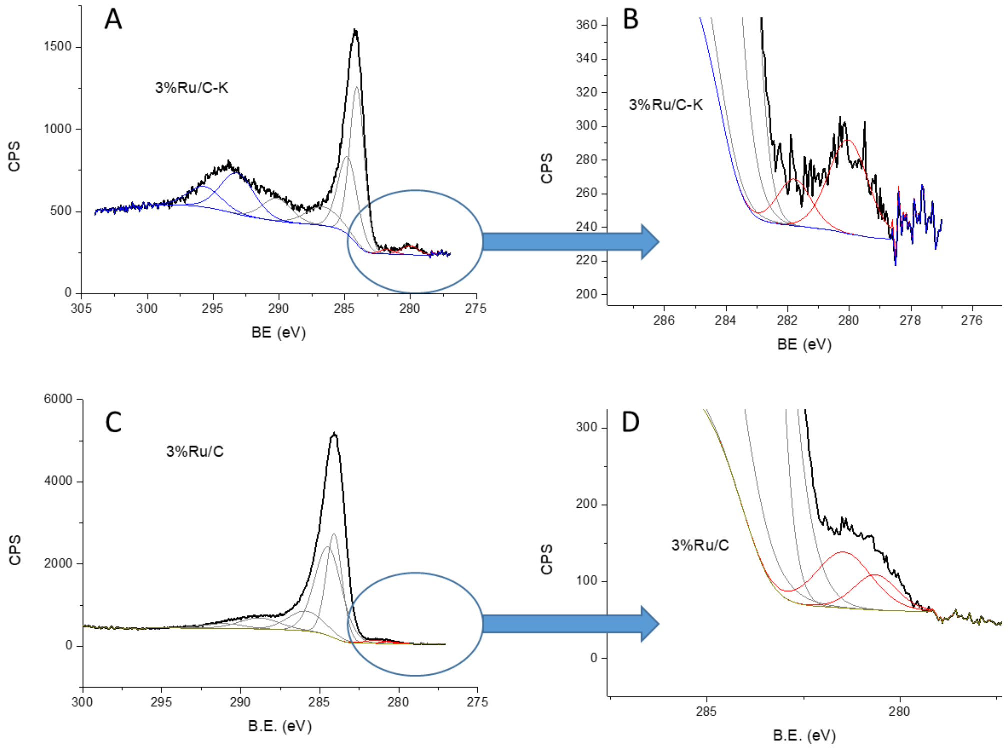

Figure S11. XPS spectra of C1s and Ru3d of reduced 3 % Ru/C-K_{450} (A,B) and 3% Ru/C (C,D) samples. Grey line corresponds to the C1s lines, blue to K2p and red to $Ru3d_{5/2}$.

Table S3. BE (eV) from the different components obtained from the deconvolution of the C1s and Ru3d$_{5/2}$ core lines in the 3 % Ru/C and the 3% Ru/C-K450 samples.

Sample	C1s (eV)	Ru3d$_{5/2}$ (eV)<br>Ru^0 RuO_2	K2p(eV)
3%Ru/C	284.5 284.4 285.8 288.6 291.0	280.6 281.5	--
3%Ru/C-K$_{450}$	284.5 284.8 286.6 290.1	280.0 281.7	293.3

Table S4. Surface composition determined by XPS of promoted and un-promoted samples.of reduced 3% Ru/C. In order to obtain a stable pellet, all samples have been diluted with silicon oxide for in situ reduction (See more details in experimental section). Under these conditions Ru3d$_{5/2}$ is not detected, being in the limit of detection.

Catalyst	C: O : (K, Cs, Na) [a]
C	n.d.
3%Ru/C	92.2: 7.7: --
3%Ru/C-K$_{450}$	88.9: 0 : 11.04
3%Ru/C-Cs$_{450}$	59.6: 36.7 : 3.59
3%Ru/C-Na$_{450}$	n.d.

[a] O1s component of silicon oxide is not included for composition analysis.

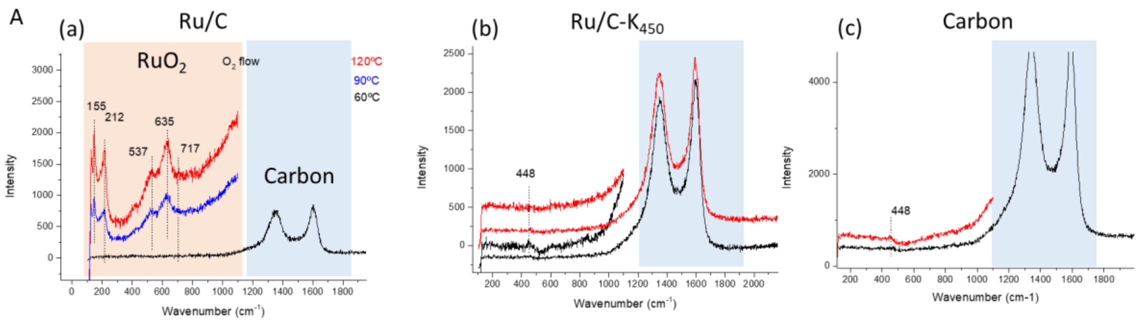

Figure S12. A) Raman spectra in O_2 flow at 60 °C (black line), 90 °C (blue line) and 120 °C (red line) on the 3% Ru/C (a), 3% Ru/C-K_{450} (b) and pure Carbon (c).

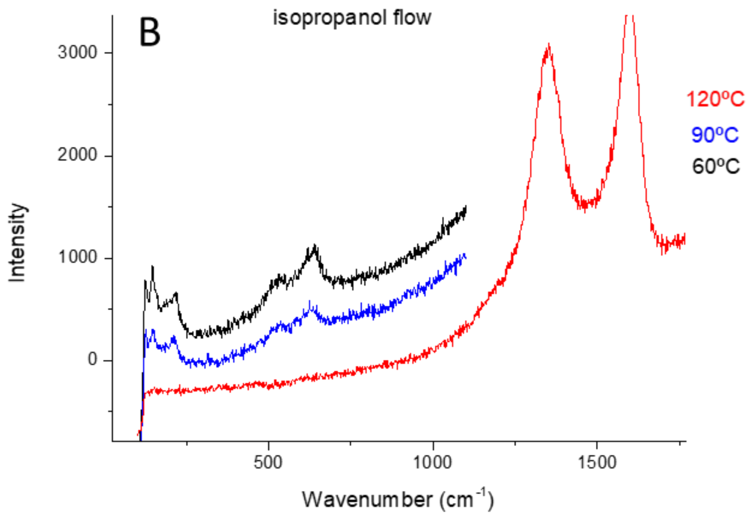

B) Raman spectra of the 3% Ru/C sample, acquired by transitioning from oxygen to isopropanol flow, coupled with a gradual temperature increase from 60 to 120 °C. The absence of all Raman bands associated with oxygen suggests their involvement in the underlying reaction mechanism.

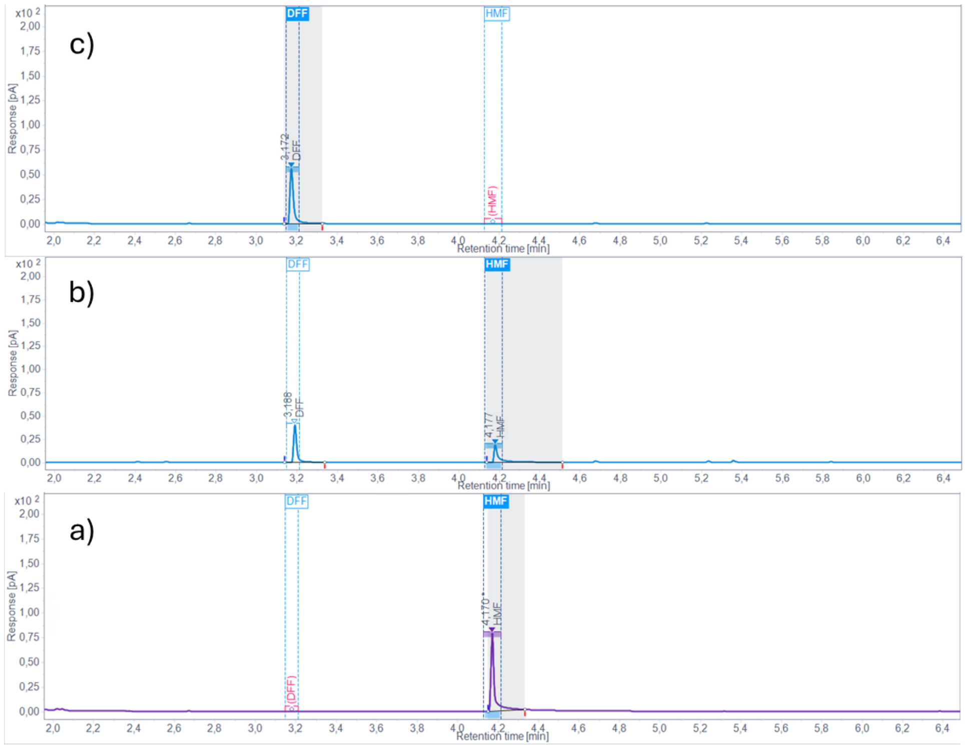

Figure S13. GC spectra of HMF conversion to DFF during the reaction at (a) 0h, (b) 30 min and (c) 3h.

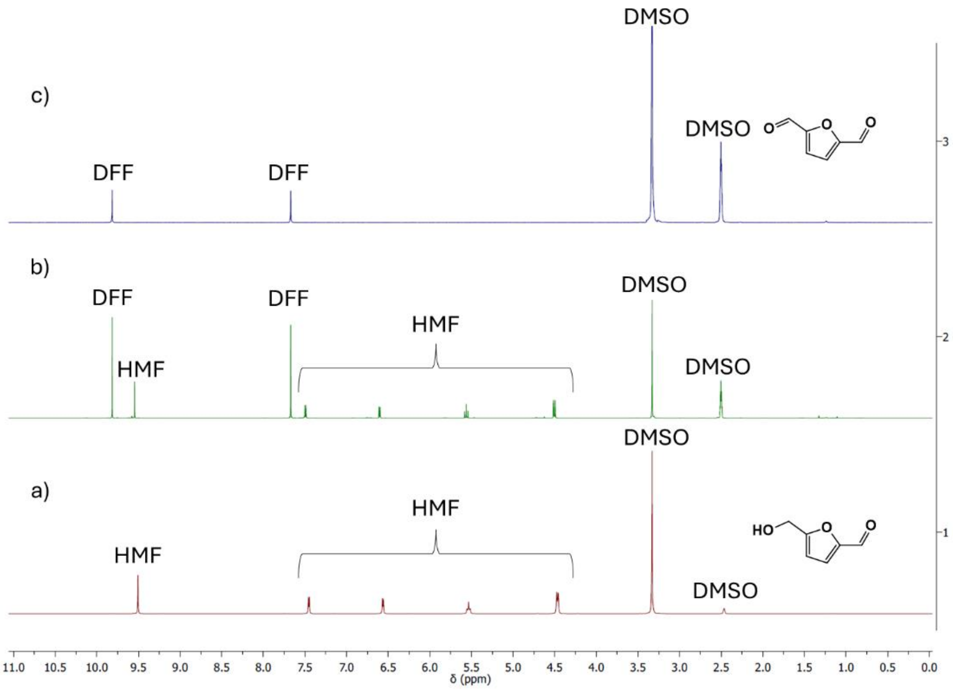

Figure S14. ^{1}H NMR spectra of the HMF conversion to DFF at (a) 0h, (b) 45 min, (c) 3h of reaction using 3% Ru/C-K_{450} catalyst.

Experimental characterization

The nanoparticle size distribution of prepared samples starting from different molar ratios of metal salt precursors were characterized by transmission scanning microscope (STEM) and energy dispersive X-ray spectroscopy (EDS) elemental mapping. TEM images of the catalyst were taken with a JEOL JEM 2100F electron microscope operating at 200 kV. Before being transferred into the TEM chamber, the samples were deposited onto a carbon-coated copper grid and then quickly moved into the vacuum evaporator.

The dispersion of Ru in the studied catalysts was estimated from the CO adsorption using the double isotherm method on a Quantachrome Autosorb-1C apparatus. Before adsorption, the samples were in situ reduced with a pure hydrogen flow (25 mL/min) at 400 °C for 2 h (ramp of 10°C/min). After reduction, the samples were degassed and then the temperature was lowered to 25°C. Following, pure CO was supplied, and the first adsorption isotherm was measured. After evacuation at 25°C, the second isotherm was taken. The amount of chemisorbed CO was obtained by subtracting the two isotherms. The dispersion of Ru was calculated from the amount of irreversibly adsorbed CO, assuming a Ru/CO stoichiometry of 1:1.

Raman spectra were recorded using a 514 nm excitation laser on a Renishaw Ramen ("Reflex") spectrometer equipped with a CCD detector. The laser power on the samples was 25 mW, and a total of five consecutive acquisitions were taken for each spectrum. The in situ studies were carried out in a Linkam CCR1000 reaction cell introducing 50 mg of catalyst, and submitting the catalyst to an air flow (20 ml/min) at increasing temperatures (60, 90 and 120 °C), while collecting spectra at each temperature. After it, the temperature was decreased to 60 °C in air flow and switched to a flow of isopropanol, obtained by bubbling Ar through a saturator of isopropanol. After stabilization, the temperature was increased gradually to 90 and 120 °C, and at each temperature several spectra were collected.

XPS studies were performed using a SPECS spectrometer with a MCD-9 detector and using a non monochromatic AlKα (1486.6eV) X-Ray source. Spectra were recorded using analyzer pass energy of 50 eV, an X-ray power of 200W and under an operating pressure of 10^{-9} mbar. During data processing of the XPS spectra, binding energy (BE) values were referenced to C1s peak (284.5 eV). Spectra treatment has been performed using the CASA software. Reduced samples were studied after in situ H_2 reduction in a 10 ml/min H_2 flow at 200 ^oC for 2h, in a high pressure catalytic reactor connected to the XPS chamber under vacuum conditions. For the in situ studies, the samples were diluted in SiO_2 in a 20:80 ratio, in order to obtain a stable pellet. Only in two cases, additional experiments without SiO_2 dilution were performed in order to get details about the $Ru3d_{5/2}$ core line.

The electron-donating effect was confirmed by monitoring UV−vis absorption of tetracyanoethylene (TCNE) molecules in the presence of 3% Ru/C, 3% Ru/C-K_{450}, 3% Ru/C-Na_{450} and 3%Ru/C-Cs_{450} catalyst. It is well-known that TCNE can accept electrons from the vicinity of electron-rich surfaces, forming e^-/TCNE complexes that exhibit a characteristic UV−vis absorption peak at ~300 nm. To perform the experiment, a solution of TCNE (0.1 mM) in acetonitrile was prepared, and measurements were conducted over varying durations until the signal reached stabilization.

The ^1H NMR spectra were recorded using a Bruker Ascend 400 (^1H 400 MHz) in DMSO at room temperature. The chemical shifts are expressed in ppm.

Spectral data:

HMF: ^1H NMR (400 MHz, DMSO) δ 9.55 (s,1H), 7.50 (d,1H), 6.61 (d,1H), 5.57 (t,1H), 4.52 (d,2H).

DFF: ^1H NMR (400 MHz, DMSO) δ 9.82 (s,2H), 7.67 (s,2H).

References

[1] Y. Y. Gorbanev, S. Kegnæs, A. Riisager, in *Top Catal*, **2011**, pp. 1318–1324.

[2] Z. Zhang, Z. Yuan, D. Tang, Y. Ren, K. Lv, B. Liu, *ChemSusChem* **2014**, *7*, 3496–3504.

[3] S. Wang, Z. Zhang, B. Liu, J. Li, *Ind Eng Chem Res* **2014**, *53*, 5820–5827.

[4] Y. Wang, B. Liu, K. Huang, Z. Zhang, *Ind Eng Chem Res* **2014**, *53*, 1313–1319.

[5] J. Artz, R. Palkovits, *ChemSusChem* **2015**, *8*, 3832–3838.

[6] J. Artz, S. Mallmann, R. Palkovits, *ChemSusChem* **2015**, *8*, 772–789.

[7] Y. Zhu, X. Liu, M. Shen, Y. Xia, M. Lu, *Catal Commun* **2015**, *63*, 21–25.

[8] Z. Yang, W. Qi, R. Su, Z. He, *Energy and Fuels* **2017**, *31*, 533–541.

[9] F. Wang, Z. Yuan, B. Liu, S. Chen, Z. Zhang, *Journal of Industrial and Engineering Chemistry* **2016**, *38*, 181–185.

[10] K. Ghosh, R. A. Molla, M. A. Iqubal, S. S. Islam, S. M. Islam, *Appl Catal A Gen* **2016**, *520*, 44–52.

[11] D. K. Mishra, J. K. Cho, Y. J. Kim, *Journal of Industrial and Engineering Chemistry* **2018**, *60*, 513–519.

[12] Y. Liu, T. Gan, Q. He, H. Zhang, X. He, H. Ji, *Ind Eng Chem Res* **2020**, *59*, 4333–4337.

[13] H. F. Ren, X. Luo, K. Zhang, Q. Cai, C. L. Liu, W. S. Dong, *Journal of Porous Materials* **2020**, *27*, 1003–1012.

[14] T. Boonyakarn, J. J. Wiesfeld, M. Asakawa, L. Chen, A. Fukuoka, E. J. M. Hensen, K. Nakajima, *ChemSusChem* **2022**, *15*, DOI 10.1002/cssc.202200059.

[15] A. Takagaki, M. Takahashi, S. Nishimura, K. Ebitani, *ACS Catal* **2011**, *1*, 1562–1565.

[16] C. A. Antonyraj, J. Jeong, B. Kim, S. Shin, S. Kim, K. Y. Lee, J. K. Cho, *Journal of Industrial and Engineering Chemistry* **2013**, *19*, 1056–1059.

[17] J. Xie, J. Nie, H. Liu, *Cuihua Xuebao/Chinese Journal of Catalysis* **2014**, *35*, 937–944.

[18] J. Nie, J. Xie, H. Liu, *J Catal* **2013**, *301*, 83–91.

[19] J. Sha, B. T. Kusema, W. J. Zhou, Z. Yan, S. Streiff, M. Pera-Titus, *Green Chemistry* **2021**, *23*, 7093–7099.

[20] Q. Ke, Y. Jin, F. Ruan, M. N. Ha, D. Li, Y. Cao, H. Wang, T. Wang, V. N. Nguyen, X. Han, X. Wang, P. Cui, P. Cui, *Green Chemistry* **2019**, *21*, DOI 10.1039/c9gc01041f.

[21] P. Pal, S. Saravanamurugan, *ChemCatChem* **2020**, *12*, DOI 10.1002/cctc.202000086.
